# Pediatric multisystem inflammatory syndrome temporally associated with SARS-CoV-2 symptoms in Iran

**DOI:** 10.2217/fvl-2021-0156

**Published:** 2021-11-26

**Authors:** Mehran Akbari, Mojtaba Didehdar, Mohsen Nazari, Davood Azadi

**Affiliations:** ^1^Department of Nursing, School of Nursing, Khomein University of Medical sciences, Khomein, 39186, Iran; ^2^Department of Medical Parasitology & Mycology, School of Medicine, Arak University of Medical Sciences, Arak, 38186, Iran; ^3^Department of Pediatrics, Imam Khomeini Hospital, Khomein university of Medical Sciences, Khomein, 39186, Iran; ^4^Department of Basic & Laboratory Sciences, School of Nursing, Khomein University of Medical Sciences, Khomein, 39186, Iran

**Keywords:** COVID-19, IVIG, PIMS-TS, SARS-CoV-2

## Abstract

**Aim:** We report two cases of pediatric patients diagnosed and treated for pediatric multisystem inflammatory syndrome temporally associated with SARS-CoV-2 (PIMS-TS) symptoms. **Materials & methods:** Two previously healthy 3- and 4-year-old boys were referred to the hospital after 5 days of 39°C fever, with symptoms such as erythema multiform in the lower extremities, irritability, refusal to eat, restlessness, lymphadenopathy, conjunctivitis and abnormal echocardiography. **Results:** After 8 days of hospitalization, the patients showed normal laboratory tests, improvement of clinical condition and were discharged from the hospital. **Conclusion:** This study raised several issues for physicians about SARS-CoV-2, its complications, diagnosis and treatment. Based on our results, pediatrics with PIMS-TS should be first screened for SARS-CoV-2, then treated with a combination of antivirals, anti-inflammatories, antibiotics and intravenous immune globulin.

SARS-CoV-2 is rapidly spreading from its origin in China to all over the world and causing a disaster in human societies in the 21st century [1,2]. The emerging coronavirus is highly contagious and infection is transmitted through droplets generated while sneezing and coughing by symptomatic and asymptomatic patients [3]. The hosts of SARS-CoV-2 vary and it infects people of all ages and genders. Furthermore, evidence shows that the symptoms of the disease caused by this virus can be diverse, ranging from mild respiratory and gastrointestinal distress to multi-organ involvement such as Kawasaki syndrome [4,5].

Throughout the world, there are several types of SARS-CoV-2 causing COVID-19. The types of SARS-CoV-2 are also classified into different categories by the CDC, which can cause different symptoms in an infected person [6,7].

Kawasaki disease (KD) is a systemic syndrome with an acute fever that affects the body’s arteries, especially the coronary arteries and is one of the leading causes of heart disease in children in industrialized countries. In general, no specific test is available to diagnose the syndrome and diagnosis is carried out based on clinical symptoms, including high 5-day-fever, changes in the mucous membranes of the lips and mouth, bilateral conjunctivitis, cervical lymphadenopathy and polymorphic eczema [8].

A possible distinguishing factor between KD and pediatric inflammatory multisystem syndrome temporally associated with SARS-CoV-2 (PIMS-TS) or multisystem inflammatory syndrome in children (MIS-C) could be the difference in platelet counts. In KD, the underlying cause of the disease is mediated by the immune system, which highlights the need to receive intravenous immune globulin (iv.Ig) drugs that compete with Fc receptors, which in turn improves symptoms. If not treated properly, it can lead to an increase in platelets. However, in PIMS-TS or MIS-C, virus-secreted inflammatory mediators invade the body to stimulate CD4^+^ cells and can lead to platelet depletion [9]. Other symptoms that can be seen in PIMS-TS include abdominal pain, diarrhea, fever and shock, which require special care of the baby [10].

Unlike adults, children were thought to be less likely to be infected with the virus, and if so, they were more likely to have asymptomatic or mild illness. Given recent data on a wide range of changes in immunological and clinical findings, a complex immune mechanism may play a role in the pathogenesis of MIS-C influenced by geographical and ethnic conditions [11].

The symptoms of MIS-C are similar to KD; however, the inflammatory storm that occurs in MIS-C is more severe than KD. Another important difference between KD and MIS-C is that approximately 5% of children with KD have heart disease, but this ratio in children with MIS-C is 2%. Therefore, treatment strategies for children with MIS-C can be associated with KD treatment [12]. According to a retrospective study case series, lung CT scan of children with MIS-C has shown pleural effusion (80%), pulmonary consolidations (73%) and ground-glass opacities (91%) [13]. In our study, ground-glass turbidity was seen according to Figure 1.

**Figure 1.**
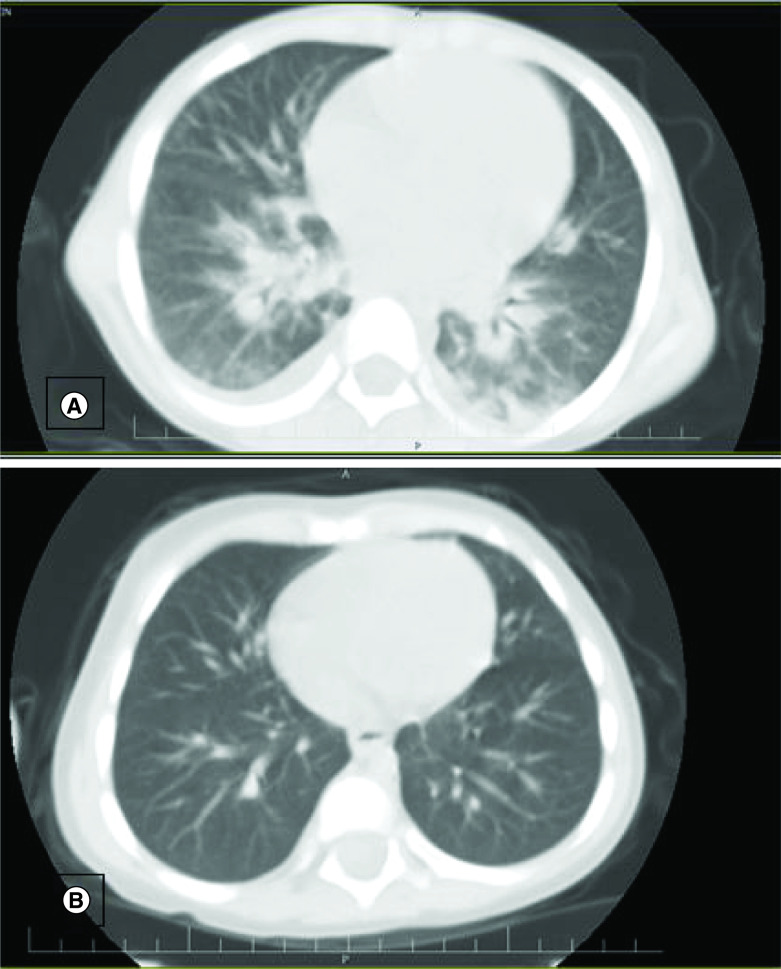
Computed tomography scans of two patients showed bilateral involvement of the lungs with sub-pleural multi lobar interstitial linear opacities and partial pleural effusion. **(A)** Case 1. **(B)** Case 2.

In the present study, we report a case series of pediatric patients with MIS associated with SARS-CoV-2 symptoms in the setting of confirmed COVID-19 infection in the city of Khomein in Iran. This study aimed to evaluate the symptoms of COVID-19 in children. This coronavirus can manifest itself in children with different symptoms than adults, which also influences its treatment following the symptoms. With proper diagnosis and treatment of PIMS-TS, serious problems such as impaired growth and development in the future, can be prevented.

## Case description

Cases: 2-, 3- and 4-year-old boys were referred to our hospital with multiple symptoms including 5 days of 39 and 39.5°C fever, respectively, irritability, refusal to eat, severe restlessness and bilateral neck lymphadenopathy.

### Case 1

A 3-year-old boy, with a weight of 13 kg. His mother also had COVID-19. Clinical examinations revealed restlessness, bilateral neck lymphadenopathy, erythema multiform in the lower extremities and his body temperature was 39°C.

### Case 2

A 4-year-old boy, who is the second child in the family and was born by cesarean delivery. His weight is 15.5 kg. Clinical examinations revealed irritability, and refusal to eat. He did not exhibit cough, congestion or rhinorrhea and had a fever of 39.5°C, with no focal signs of infection.

On the 3rd day of fever, they showed an erythematous non-pruritic rash on their body and conjunctivitis in both eyes. They did not exhibit congestion, rhinorrhea, cough, ear discharge, vomiting, diarrhea, icterus and seizure. Clinical examination revealed that the patients had 39 and 39.5°C fever with no focal signs of infection. The patients had received several doses of antibiotics and antipyretic drugs (acetaminophen 20 mg, vancomycin 200 mg, remdesivir 75 mg, enoxaparin 75 mg and dexamethasone 2.5 mg) within 5 days before being admitted to the hospital. However, the patients’ fever did not decrease and they were finally admitted to the hospital on the 5th day with the mentioned symptoms.

Laboratory tests including blood and urine analyses with culture were performed but all of them had negative results. Echocardiography of two patients showed that the left ventricular has a normal function, the left main coronary artery is 3.0 mm and left anterior descending artery is 2.37 mm, but proximal right coronary artery is 1.8 mm. As a result of these symptoms, PIMS-TS symptoms was diagnosed. After admission to the hospital, because of the unknown origin fever and the fact that we are in the COVID-19 pandemic, the patients were evaluated for SARS-CoV-2 infection. Accordingly, the RT-PCR of nasopharynx swab from two patients was carried out and the results were positive. Moreover, the results of chest computed tomography (CT) scans of two patients showed bilateral involvement of the lungs with sub-pleural multi-lobar interstitial linear opacities and partial-pleural effusion (Figure 1), and also results of blood parameters including; CRP: 2–3^+^; erythrocyte sedimentation rate (ESR): 65–115; and d-dimer: >10,000.0 ng/dl demonstrated that the patients had COVID-19 (Table 1). Additionally, parents of patients were analyzed for COVID-19 and both of them were positive.

**Table 1.** Results of laboratory parameters of patients before and after treatment.

	Laboratory parameters	Urine culture (24 h)	Blood culture (72 h)	d-dimer (ng/ml)	CRP	ESR (mm/h)	WBC (/μl)	RBC (/μl)	Hemoglobin (Hb) (g/dl)	PLT (/μl)	Urea (mg/dl)	Creatinine (mg/dl)	SARS-CoV-2 RT-PCR	SARS-CoV-2-IgG
Case 1	Before treatment	No growth	No growth	>10,000	3+	65	6.9 × 10^3^	4.11 × 10^3^	11.6	140 × 10^3^	45	0.75	+	0.1
	After treatment	No growth	No growth	400	1+	20	10.1 × 10^3^	4.07 × 10^3^	11.2	616 × 10^3^	25	0.78	-	1.9
Case 2	Before treatment	No growth	No growth	>10,000	2+	115	6.6 × 10^3^	3.32 × 10^3^	8.5	161 × 10^3^	32	0.46	+	0.1
	After treatment	No growth	No growth	200	0	32	10.1 × 10^3^	4.01 × 10^3^	10.2	459 × 10^3^	32	0.46	-	2.1

ESR: Erythrocyte sedimentation rate; CRP: C-reactive protein; RBC: Red blood cell; WBC: White blood cell; PLT: Platelet count test; RT-PCR: Reverse transcription polymerase chain reaction.

Initially, treatment was started for both children to control fever with apotel, ceftriaxone and vancomycin. Moreover, due to the diagnosis of PIMS-TS symptoms, the vitamin C, zinc vitamin B (5 unit), iv.Ig vial (15 g) methylprednisolone (7.5 mg/kg), aspirin (300 mg) and ceftriaxone (500 mg) were prescribed to the patients as a treatment to reduce the rate of inflammation and reduce the ascending course of the disease. Finally, due to the involvement of the lungs in favor of SARS-CoV 2, remdesivir was used for both patients. After 2 days of medication, the d-dimer and CRP levels decreased to <500 ng/dl and 2, respectively. Moreover, the heart rate and arterial oxygen saturation of the patients reached 110 and 98%, respectively (Table 1). The patients were hospitalized for 8 days and following the improvement of rash and conjunctivitis and other normal vital signs, they were discharged from the hospital.

## Discussion

Several reports from many parts of the world have revealed that COVID-19 infection can be presented in different symptoms and disorders in children [14,15]. Due to this issue, through the recent months, researchers have focused on definitive clinical and laboratory findings of this disease in different age groups, especially children [16]. In a study conducted by Toubiana *et al.* in Paris, the onset of Kawasaki syndrome in children with SARS-CoV-2 has been reported [17].

Kawasaki syndrome has sporadically been reported in association with COVID-19 infection from a few centers around the world [17,18]. In this study, for the first time, we report two children with multi-organ dysfunctional syndrome in children with COVID-19 symptoms as well as Kawasaki-like symptoms. They were referred to Imam Khomeini hospital located in Khomein. Our patients tolerated 39°C fever for 5 days, they had also erythema multiform in the lower extremities, irritability, refusal to eat, restlessness, lymphadenopathy, conjunctivitis and abnormal echocardiography. COVID-19 was diagnosed in the patients through assessing laboratory parameters, including the high level of CRP, d-dimer and ESR. Although bilateral involvement of the lungs was identified in lung CT, the results of the test for RT-PCR nasopharyngeal swab were negative. Based on this condition, Kawasaki syndrome was diagnosed in patients and then treated.

This study showed that when the COVID-19 enters the body, it can cause various complications due to its specific clinical manifestations, including PIMS-TS symptoms. Furthermore, the results of this study and other studies showed that unknown origin and persistent fever in children with the appearance of multi-organ symptoms can be an important sign of COVID-19 infection in children [18,19]. In addition, the results of this study showed that, due to the simultaneous diagnosis of PIMS-TS associated with SARS-CoV-2 infection, which shows symptoms similar to Kawasaki syndrome, patients undergo a combination of viral and systemic treatment, which leads to their recovery. Therefore, the use of antiviral, anti-inflammatory drugs, as well as the use of iv.Ig, is recommended for such children who are infected with COVID-19 and present to the hospital with symptoms such as PIMS-TS.

## Conclusion

This study can be useful to provide better treatment team care in children with COVID-19. Because the symptoms of COVID-19 are changing every day and the knowledge about the diagnosis of this virus is expanding, the need for appropriate and accurate treatment is necessary. Moreover, the present knowledge about the COVID-19 pandemic is limited and up to today, children are considered as the population being minimally affected by SARS-CoV-2 infection. One of the limitations is the lack of a specific diagnostic kit that can show the high specificity and sensitivity of this virus. It can be said that if the SARS-CoV-2 is not definitively diagnosed, the treatment of this disease will be delayed and it can endanger the patient’s life. At present, diagnostic corpses may report a false-negative test result, even if the patient is positive. Given that COVID-19 is currently being developed in the world, this study aims to show the effects of this virus and how to provide appropriate treatment.

**Executive summary**

- Considering that SARS-CoV-2 can show different clinical symptoms in children than adults, in this present study, we report two cases who were diagnosed and treated for pediatric multisystem inflammatory syndrome temporally associated with SARS-CoV-2 (PIMS-TS) symptoms.
- The purpose of this case report is to diagnose and treat children with PIMS-TS to avoid serious complications that may occur in the future. The children were diagnosed to have (PIMS-TS) symptoms and then, they were treated with remdesivir, vitamin C, intravenous immune globulin, methylprednisolone and ceftriaxone.
- This research can be useful to provide better treatment team care in children with COVID-19. Because the coronavirus is still changing, it can present itself daily with a variety of symptoms that require newer treatments, especially when it comes into contact with PIMS and Kawasaki disease.
